# An Exploration of the Association Between Occupational Stress and Fibromyalgia Among Healthcare Professionals: A Cross-Sectional Study

**DOI:** 10.31138/mjr.120824.asr

**Published:** 2025-06-30

**Authors:** Asma Alneyadi, Noor Yousif Alnuaimi, Hajar Mohammed Almansoori, Sara Hasan Alhosani, Shammah Almemari, Muhammad Jawad Hashim, Shamma Ahmad Al Nokhatha

**Affiliations:** 1 Internal Medicine;; 2 College of Medicine and Health Sciences;; 3 Department of Family Medicine; 4 Rheumatology, Tawam Hospital, Al Ain, United Arab Emirates

**Keywords:** occupational stress, fibromyalgia, healthcare professionals, gender disparities, Abu Dhabi, Surveys, questionnaires

## Abstract

**Introduction:**

High levels of stress among healthcare workers may impact the quality of care provided to patients. Chronic stress can lead to conditions such as fatigue and fibromyalgia. We aimed to assess stress levels and identify healthcare workers at risk for occupational burnout and fibromyalgia.

**Methods:**

We conducted a cross-sectional study among healthcare professionals in the Abu Dhabi region. Physicians, nurses and other healthcare workers were invited to take part in an anonymous survey via email. The questionnaire included validated scales for workplace stress as well as for diagnostic symptoms for fibromyalgia.

**Results:**

Among the 254 respondents, the majority were females (73.2%) aged 20 to 59 years. Females reported higher stress levels than males (mean scores 6.7 vs 5.7; on a scale of 0 to 10), and physicians reported higher stress than nurses (mean scores 7.2 vs 5.8). More than half of the professionals were considering quitting due to work-related stress; highest among of resident physicians (65%). Fibromyalgia criteria were met by 28.3% of respondents, predominately females, with a significant association observed between higher stress levels and a fibromyalgia diagnosis.

**Conclusions:**

High levels of stress were reported by healthcare professionals, especially women. Stress was associated with fatigue, fibromyalgia, and burnout leading to the intention of leaving clinical work. Workplace changes and stress reduction support programs are needed urgently to protect this vital workforce.

## INTRODUCTION

Occupational stress has been extensively reported as a significant risk factor for various physical and mental health conditions.^1^ Stress can have a profound impact on the body, affecting every organ-system and ultimately leading to illness.^2^ One such condition is Fibromyalgia (FM), which is characterised by a range of symptoms that primarily include chronic, widespread musculoskeletal pain, as well as allodynia and hyperalgesia.^3,4^ The condition is distinguished from other rheumatological conditions, by the absence of tissue inflammation and end-organ damage.^5^ Individuals suffering from fibromyalgia often experience physical and mental fatigue, intolerance to exertion, non-restorative sleep, and additional functional complaints, such as gastrointestinal issues. This condition significantly overlaps with other functional somatic syndromes, particularly chronic fatigue syndrome, and is notably more prevalent in women.^6,7^

Recent studies suggest that continuous exposure to stressful events and poor mental health can contribute to the development of central sensitisation, a key mechanism underlying fibromyalgia pathogenesis.^2^ Healthcare workers are among the populations at the highest risk of experiencing occupational stress and its adverse consequences.^8^ Factors such as being female, single, younger in age, and living alone can contribute to higher incidences of mental health problems among healthcare workers.^9^ According to (Joshi et al.2022), the reported prevalence of occupation stress varies from 27% to 87.4%.^10^ A recent meta-analysis by Girma et al. (2021) has reported an occupational stress prevalence of 52.5% among healthcare workers.^11^ Additionally, high workloads, uncooperative colleagues, and fear of making mistakes have been identified as contributing factors to stress in this population.^9^ There are work, individual, and social-related factors that ultimately lead to an increased risk of burnout and occupational stress.^10^ Work-related factors include long working hours, working in unsupportive environments, limited resources, and making life-saving decisions. Personal factors contributing to stress include poor time management, poor sleep hygiene, and low socioeconomic status.^10^ A cross-sectional study conducted in the Middle East region found that over 50% of healthcare workers experienced depression, anxiety, and stress.^12^ Further, healthcare professionals, particularly those working in Saudi Arabia and Pakistan, face unique challenges leading to high levels of occupational stress and the development of associated conditions such as fibromyalgia.^13,14^ Therefore, providing adequate psychological support and improving overall mental well-being for healthcare professionals is crucial especially at times of high patient loads.^15^

Despite extensive research linking stress to various physical conditions, including fibromyalgia, there is a notable absence of studies specifically investigating the prevalence and risk factors of fibromyalgia among healthcare workers. Existing literature highlights the high incidence of occupational stress in healthcare professionals and its various contributing factors, such as high workloads, unsupportive work environments, and personal challenges. However, understanding the prevalence and impact of this relationship is essential for developing effective interventions to support the well-being of healthcare professionals and mitigate the burden of fibromyalgia in this population.

Herein, we aim to identify healthcare workers at risk of occupational stress and fibromyalgia, and determine the factors that may increase susceptibility to these conditions.

## METHODS

This cross-sectional observational study was conducted in the health regions of Abu Dhabi city, at three tertiary hospitals; Tawam, Shaikh Shakhbout Medical City, and Shaikh Khalifa Medical City hospitals. Participants were healthcare professionals (nurses, residents and attending physicians (general practitioner, specialists and consultants) who voluntarily agreed to participate and completed an anonymous survey between November 2023 and February 2024. Data were anonymized to ensure confidentiality. The overall response rate based on the total workforce was 4.5%. All doctors and nurses who worked in the aforementioned Abu Dhabi regions were contacted by email with an anonymous questionnaire about occupational stress. The questionnaire was designed by integrating demographic questions, validated diagnostic criteria, and participants’ suggested strategies to reduce stress. The initial draft was reviewed by the coauthors to ensure content validity. A pretest was conducted with a small sample of participants to assess the clarity and comprehensibility, then feedback was used to refine the questionnaire for better clarity and flow. The final questionnaire version was reviewed again by the coauthors to ensure internal consistency and coherence across the sections. To address specific aspects of occupational stress and fibromyalgia, in addition to the validated scale used to calculate workplace stress and fibromyalgia, certain sections of the questionnaire were self-developed, and is provided as **Supplementary File 1**.

The self-administered questionnaire assessed various aspects. It included items related to sociodemographic characteristics, comorbidities, career level, clinical departments, and working shifts. Occupational stress levels were measured using the Workplace Stress Scale (WSS), which evaluates workplace-related stress. The WSS consists of eight items that describe the respondent’s feelings toward their job, following a five-point Likert scale format, ranging from never (scored 1) to very often (scored 5).^16–18^ Higher scores indicate higher stress levels, with a total score of 15 or lower indicating chilled out, 16–20 indicating low stress, 21–25 indicating moderate stress, 26–30 indicating severe stress, and a score of more than 30 considered to be a potentially dangerous stress level.^16^ The impact of stress on quality of life, with a focus on fibromyalgia, was also assessed. Fibromyalgia was defined by diagnostic criteria that included questions on the Symptom Severity Score (SSS) and the Widespread Pain Index (WPI). The SSS has two components. The first component includes three major symptoms (fatigue, trouble thinking or remembering, waking up tired or un-refreshed) which are coded 0 (no problem), 1 (mild), 2 (moderate), or 3 (severe). The second component includes somatic symptoms that have been present for at least three months, with 0 (not present), and 1 (present) (e.g. pain or cramps in lower abdomen, depression, headache), with a maximum score of 3. The WPI includes 19 non-articular pain sites where the respondents reported experiencing pain in the past week. A positive response was identified when participants recorded a WPI score of ≥7/19 for pain sites and SSS score of ≥5. Alternatively, a positive response was also recorded if participants achieved a WPI score between 3-6/19 and an SSS score of ≥9, with Symptoms persisting at a similar level for at least 3 months, and the absence of any other disorder that could explain the pain.^19,20^ Respondents also provided proposed coping strategies. At the time of the survey, 5,668 healthcare professionals worked in the centres described above, and a response rate of 4.5% was obtained.

### Statistical analysis

The base line characteristics were represented in terms of frequency and percentage for categorical data and mean±SD for continuous variables. Association of categorical variables were evaluated using chi square test. A linear regression analysis was conducted to find significant predictors for stress. A logistic regression analysis was done to find out the risk factors associated with fibromyalgia. Data were analysed using Jamovi statistical software which uses the R statistical package. An alpha level of 0.05 was pre-specified as the cut-off value for statistical significance. Missing values were not imputed.

## RESULTS

A total of 254 healthcare professionals participated in the study, with a majority being female (73.2%) and nearly all respondents aged between 20 to 59 years (98.8%). **Table 1** shows the baseline characteristics of participants including age, gender, ethnicity and other demographic parameters. The age distribution showed a larger proportion of younger individuals (20–29) among physicians (50.4%) compared to nurses (7.6%). The age groups 30 - 39 years and 40 - 49 years were more among nurses (*p* < 0.001). Gender distribution was the same among nurses (73.2% females) and physicians (73.2% females). Majority of healthcare workers were females 186 (73.2%) as compared to males, 68 (26.8%). Marital status showed a significant difference in proportion between nurses and physicians with a higher proportion of single physicians (48.8%) as compared to nurses (17.6%) (*p* < 0.001).

**Table 1.** Baseline characteristics of participants (n=254).

**Characteristic**	**Nurses N (%)**	**Physicians N (%)**	**All N (%)**	**p-value**
**Age**				
20 – 29	10 (7.6)	62 (50.4)	72 (28.3)	
30 – 39	64 (48.9)	31 (25.2)	95 (37.4)	
40 – 49	40 (30.5)	15 (12.2)	55 (21.7)	< 0.001
50 – 59	16 (12.2)	13 (10.6)	29 (11.4)	
60+	1 (0.8)	2 (1.6)	3 (1.2)	
**Gender**				
Female	96 (73.3)	90 (73.2)	186 (73.2)	
Male	35 (26.7)	33 (26.8)	68 (26.8)	0.98
**Marital status**				
Single	23 (17.6)	59 (48.8)	82 (32.3)	
Married	107 (81.7)	60 (48.8)	167 (65.7)	
Divorced	0 (0.0)	4 (3.3)	4 (1.6)	< 0.001
Widow/widower	1 (0.8)	0 (0.0)	1 (0.4)	
**Nationality**				
Emirati	8 (6.1)	74 (60.2)	82 (32.3)	
Non-Emirati	123 (93.9)	49 (39.8)	172 (67.7)	< 0.001
**Ethnicity**				
Arab	30 (22.9)	101 (82.1)	131 (51.6)	
Asian	88 (67.2)	18 (14.6)	106 (41.7)	
Black	1 (0.8)	2 (1.6)	3 (1.2)	
White	3 (2.3)	2 (1.6)	5 (2.0)	< 0.001
Other	9 (6.9)	0 (0.0)	9 (3.5)	
**Financial dependents**				
4 or fewer	75 (57)	96 (78)	171 (67)	
5 or more	56 (43)	27 (22)	83 (33)	< 0.001
**Clinical department**				
Medical	105 (80.2)	117 (95.1)	222 (87.4)	
Surgical	26 (19.8)	6 (4.9)	32 (12.6)	< 0.001
**Work hours**				
Shifts	76 (58.0)	68 (55.3)	144 (56.7)	
Fixed hours	55 (42.0)	55 (44.7)	110 (43.3)	0.66
**Years in Abu Dhabi**				
< 5	36 (27.5)	71 (57.7)	107 (42.1)	
5 – 10	34 (26.0)	21 (17.1)	55 (21.7)	
10 – 15	21 (16.0)	17 (13.8)	38 (15.0)	
15+	40 (30.5)	14 (11.4)	54 (21.3)	< 0.001

Emiratis were more among physicians (60.2%) as compared to nurses (6.1%) (p<0.001). Similarly, Arabs constituted a majority among physicians (82.1%) compared to nurses (22.9%). Nurses were more likely to be of non-Arab Asian ethnicity (67.2 %) and in physicians they were 14.6 % (p<0.001). Most of the physicians were in the Medicine department (95.1%) as compared to nurses (80.2%). A very small number of physicians who participated in the study were in surgical departments (4.9%) as compared to nurses (19.8%).

**Figure 1.**
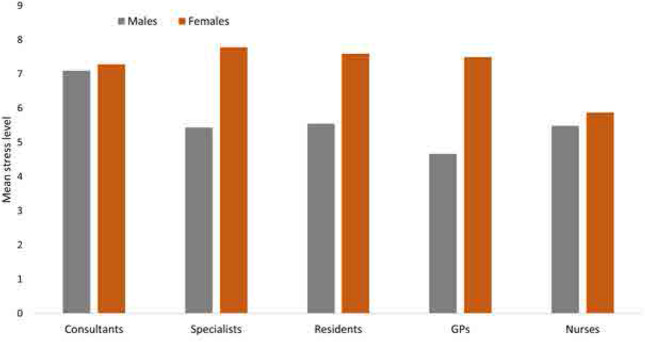
Self-reported stress levels among healthcare professionals (n = 254). p < 0.001, one-way ANOVA.

**Figure 2.**
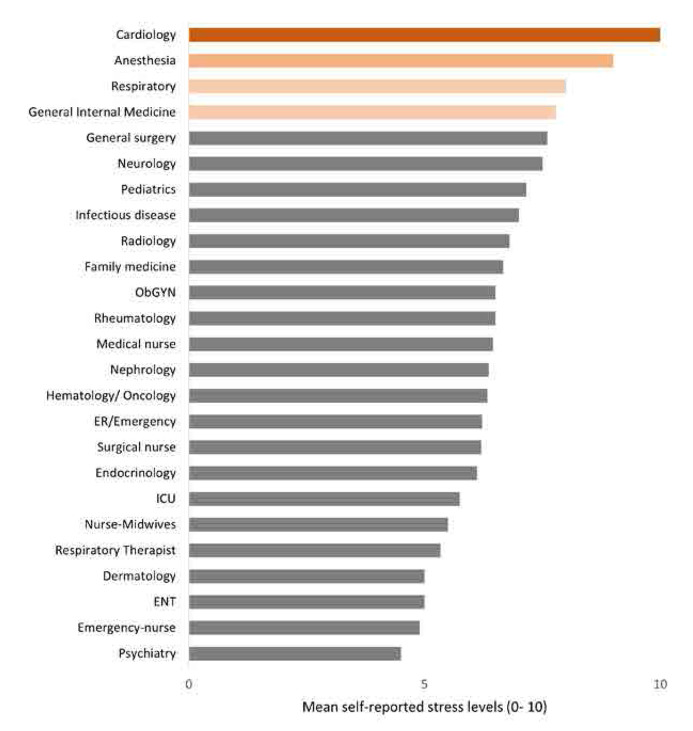
Self-reported stress levels in different clinical departments.

The mean self-reported stress level was 6.4 (SD 2.2) on a scale of 0 to 10 (**Figure 1**). Nurses reported lower stress levels compared to consultant physicians, with mean scores of 5.7 and 7.2, respectively (p < 0.001). Certain clinical departments were associated with higher levels of reported stress (**Figure 2**). **Table 2** represents the mean stress score across various parameters for nurses, attending physicians, and resident physicians. The mean stress showed a significant difference across age groups (*p* < 0.01). Attending physicians reported higher stress (7.3±2.2) as compared to nurses (6.5 ± 1.7). Gender also showed a significant difference in terms of mean stress level (p=0.002). Female showed higher stress (attending physicians (7.5±1.5), resident physicians (7.6±1.7), and nurses (5.8±2.2) than males (attending physicians (6.0±1.9), resident physicians (5.5±2.0) & nurses (5.4±2.6). Marital status also indicated a significant difference in stress levels (p=0.004) among the 3 groups. Working hours showed a significant mean difference among the groups (*p* = 0.018).

**Table 2.** Self-reported stress among healthcare professionals on a scale of 0–10.

**Characteristic**	**Attending physicians Stress**	**Resident physicians Stress**	**Nurses Stress**	**All Stress**	**p value**
	*mean (SD)*	*mean (SD)*	*mean (SD)*	*mean (SD)*	
**Age**					
20 – 29	7.5 (2.0)	7.3 (1.6)	5.9 (2.1)	7.1 (1.8)	
30 – 39	6.8 (1.5)	6.1 (3.3)	5.3 (2.3)	5.7 (2.3)	
40 – 49	7.1 (1.7)	10	6.2 (2.3)	6.5 (2.2)	
50 – 59	7.3 (2.2)	-	6.5 (1.7)	6.8 (1.9)	0.01
60+	5.0 (1.4)	-	1.0 (-)	3.6 (2.5)	
**Gender**					
Female	7.5 (1.5)	7.6 (1.7)	5.8 (2.2)	6.7 (2.1)	
Male	6.0 (1.9)	5.5 (2.0)	5.4 (2.6)	5.6 (2.3)	0.002
**Marital status**					
Single	7.7 (1.3)	7.0 (2.1)	6.1 (2.1)	7.0 (2.0)	
Married	6.4 (1.9)	7.4 (1.7)	5.7 (2.4)	6.1 (2.3)	
Divorced	7.5 (0.7)	7.5 (0.7)	-	7.5 (0.5)	0.004
**Nationality**					
Emirati	7.0 (1.6)	7.4 (1.9)	5.6 (2.5)	7.1 (1.9)	
Non-Emirati	6.9 (2.0)	6.9 (2.0)	5.7 (2.3)	6.1 (2.3)	< 0.001
**Ethnicity**					
Arab	7.0 (1.6)	7.1 (1.9)	5.7 (2.3)	6.7 (2.0)	
Asian	6.5 (2.2)	9.2 (0.9)	5.8 (2.3)	6.0 (2.3)	
Black	8.0 (0.0)	-	7.0 (-)	7.6 (0.5)	
White	8.0 (1.4)	-	6.3 (0.5)	7.0 (1.2)	0.024
Other	-	-	4.6 (2.7)	4.6 (2.7)	
**Financial dependents**					
4 or fewer	7.1 (1.8)	7.3 (1.8)	5.7 (2.2)	6.5 (2.1)	
5 or more	6.7 (1.7)	6.9 (2.5)	5.8 (2.4)	6.1 (2.3)	0.15
**Clinical department**					
Surgical	7.5 (1.7)	8.5 (0.7)	5.0 (2.4)	5.5 (2.5)	
Medical	6.9 (1.8)	7.2 (1.9)	5.9 (2.3)	6.5 (2.1)	0.047
**Work hours**					
Fixed hours	7.1 (1.5)	6.5 (2.1)	5.2 (2.1)	6.0 (2.1)	
Shifts	6.8 (2.1)	7.6 (1.7)	6.1 (2.4)	6.7 (2.2)	0.018

p-values are for comparison within the All column only using one-way ANOVA.

* indicates a statistically significant difference

From the analysis, the prevalence of occupational stress varied across categories: 47.6% of participants reported moderate stress, 29.1% severe stress, and 6.7% experienced potentially dangerous stress levels. Only a small proportion of healthcare professionals reported low stress (14.6%) or no stress (‘chilled out’ status at 2.0%).

A linear regression analysis of significant factors affecting stress was conducted. From the analysis, it was observed that the intercept can significantly predict the outcome, and it is different from zero (Estimate = 7.306 p<0.001), which indicates the baseline level. We found that gender is a significant predictor of stress score, with females showing higher stress as compared to males (Estimate = 1.249 p<0.001). Age groups were non-significant, except that a borderline effect in 30–39 age group (p=0.069) as compared to 20–29 groups.

Marital status found to be no effect in stress score in our analysis. Career status shows that nurses have significantly higher stress as compared to consultants (Estimate = 1.922 p<0.001). Shift works schedules can predict higher stress compared to fixed hours (Estimate = 0.7231 p= 0.01). From the smoking status, it was observed that ex-smokers (Estimate = 1.8015 p = 0.012) have significant higher stress as compared to nonsmokers.

**Figure 3.**
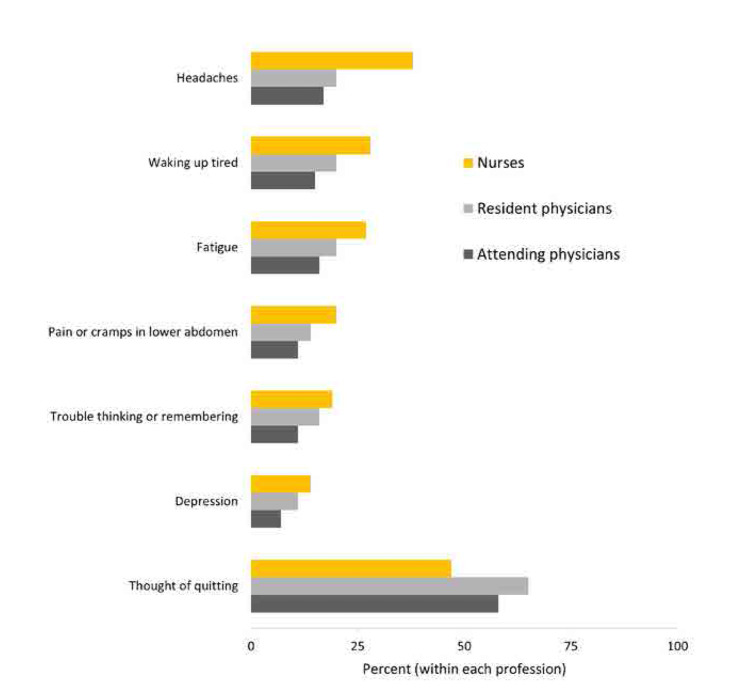
Prevalence of reside. n = 254, consisting of 131 nurses, 66 residents, and 57 attending physicians in active clinical practice.

The comparative prevalence of health conditions among healthcare professionals is illustrated in **Figure 3**. These individuals were also more likely to perceive a negative impact of stress on their personal lives and to consider quitting their jobs.

**Figure 4.**
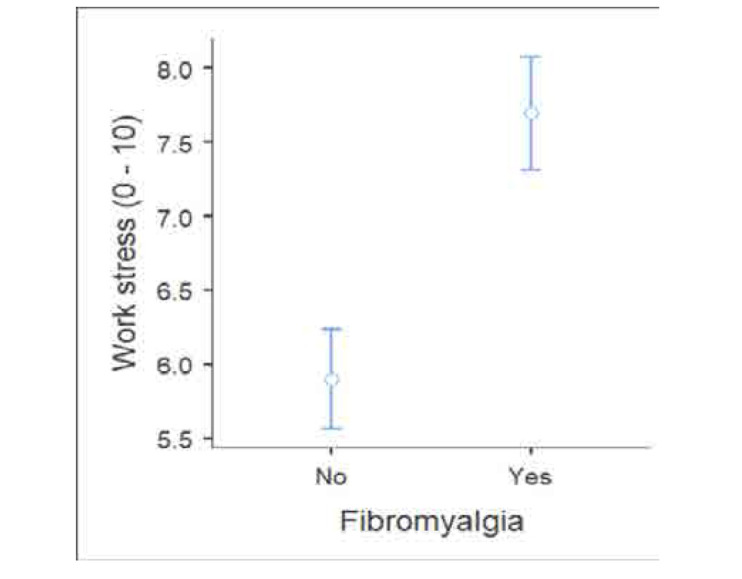
The relation between stress and fibromyalgia.

The elevated stress levels were significantly correlated with fibromyalgia (**Figure 4**). A total of 28.3% (n=72) healthcare professionals met the criteria of fibromyalgia. The distribution of fibromyalgia with comorbidities among healthcare professionals is detailed in **Table 3**, which compares those diagnosed with fibromyalgia to those without the condition. Nurses had the lowest prevalence of fibromyalgia symptoms, while resident physicians had the highest. The table also indicates several comorbidities commonly linked to stress including anxiety, migraine, and irritable bowel syndrome among those diagnosed with fibromyalgia, underscoring the varied symptomatology associated with fibromyalgia in healthcare workers and providing insight into how the condition affects different professional roles within the healthcare setting.

**Table 3.** Demographic characteristics and comorbidities among healthcare workers with and without fibromyalgia.

**Characteristic**	**With fibromyalgia**	**Without fibromyalgia**
	*N (%)*	*N (%)*
**Age**		
20 – 29	25 (35)	47 (65)
30 – 39	25 (26)	70 (74)
40 – 49	17 (31)	38 (69)
50 – 59	5 (17)	24 (83)
60+	0 (0)	3 (100)
**Gender**		
Female	64 (34)	122 (66)
Male	8 (12)	60 (88)
**Profession**		
Nurses	32 (24)	99 (76)
Residents	22 (33)	44 (67)
Attending physicians	18 (32)	39 (68)
Smoking	4 (44)	5 (56)
**Comorbidities**		
Anxiety	23 (51)	22 (49)
Depression	8 (47)	9 (53)
Diabetes	4 (22)	14 (78)
Hypothyroidism	7 (37)	12 (63)
Obesity	14 (37)	24 (63)
Irritable bowel syndrome	22 (50)	22 (50)
Migraine	23 (55)	19 (45)
Vitamin D deficiency	27 (38)	45 (63)
Autoimmune disease	4 (36)	7 (64)

Percentages are within each row.

**Figure 5.**
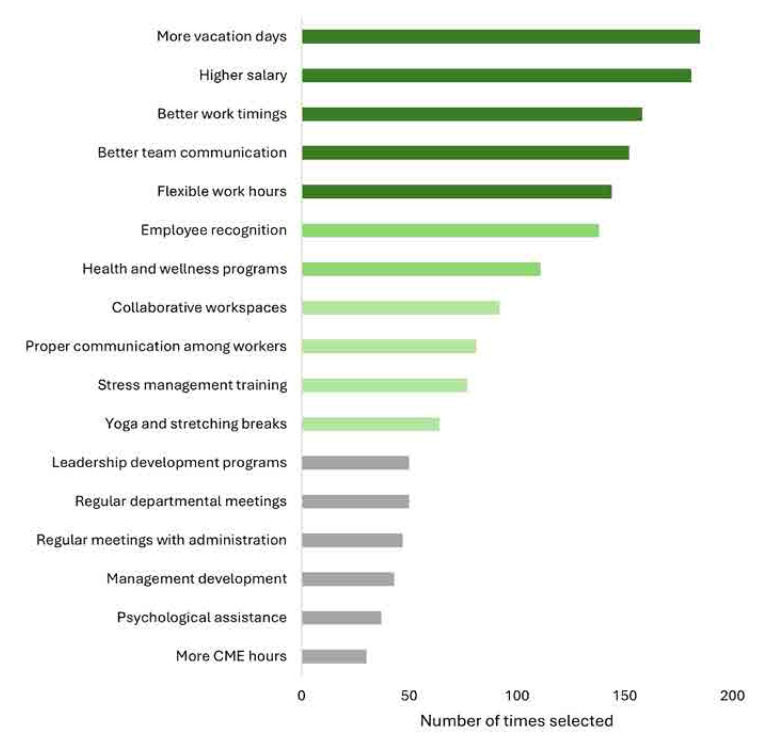
Recommendations to reduce stress. Each respondent could select more than one choice. N = 254 healthcare professionals.

Logistic regression analysis results identified significant predictors of fibromyalgia among healthcare professionals. From the analysis, it was observed that gender significantly impact the outcome. Females show higher odds (OR= 5.1341, 95% CI 1.77–14.83 p=0.003) as compared to males, which indicates females are having more chances to face fibromyalgia as compared to male. Age categories had no significant impact on the outcome. All the p values in age categories show non-significant p values. Similarly, marital status, career level and ethnicity had no impact on the outcome in our analysis. However, work turn (shift vs fixed hours) shows a significant impact on outcome. Shift workers having higher odds (OR=2.18 95% CI 1.08–4.23, p = 0.029) as compared to fixed hour workers. Lastly smoking status has significant impact on the outcome. Smokers show significant higher odds (OR = 5.607 95% CI 1.02–30.78 p = 0.047) for fibromyalgia as compared to nonsmokers. The logistic regression analysis provided a significant overview of demographic and lifestyle factors that affect fibromyalgia risk, which includes gender, work shift, and smoking status. Supplementary File 2 The respondents provided a range of suggestions to reduce occupational stress in healthcare settings. These suggestions, shown in **Figure 5**, included offering more annual vacation, flexible work hours, improving communication, and implementing stress management training.

## DISCUSSION

The study reveals a high prevalence of occupational stress among healthcare professionals in Abu Dhabi with 83.5% experiencing moderate stress or higher. The prevalence aligns with previous findings that reported rates ranging from 27% to 87.4%^10,21^ and a meta-analysis showing an overall prevalence of 52.5% among healthcare workers.^11^ The high levels of moderate to severe stress observed in this study are consistent with the demanding nature of healthcare profession, especially for specific groups such as females, those in high-stakes specialties like Cardiology and Anaesthesia, and shift workers, who all reported significantly elevated stress levels. This observation is further supported by studies like Nordin et al. (2022),^9^ which identified workload, shift work, and gender as key factors contributing to increased stress in healthcare settings.

In our study, 28.3% of healthcare professionals met the criteria of fibromyalgia, with a striking predominance of females (88.9%). This prevalence is significantly higher than that reported in general population studies, such as those by Branco et al. (2010) conducted in Europe, where the overall prevalence was estimated at around 4.7%.^22^ A study among healthcare professionals in Saudi Arabia during the COVID-19 pandemic reported a prevalence of nearly 20%.^23^

The relation between high-stress environments and fibromyalgia was high, further supported by previous studies that emphasise chronic stress is associated with fibromyalgia, which is characterised by central nervous system pain amplification, along with symptoms such as fatigue, memory problems, sleep and mood disturbances.^24^ Beiner et al. (2023) also reported that chronic stress plays a critical role in the onset and exacerbation of fibromyalgia symptoms.^25^ These findings highlight the significant impact of occupational stress on healthcare professionals and underscore the importance of examining specific factors that contribute to such high prevalence rates in this population. The diverse sample population in this study, which includes various healthcare roles and demographics, strengthens the generalizability of the findings. Additionally, the use of robust statistical analyses, including logistic regression, enhances the reliability of the results. For instance, the logistic regression analysis identified female gender and working in shifts as significant predictors of fibromyalgia, with an odds ratio of 2.5 (p < 0.05) for females and a similarly significant impact for shift workers, indicating the strong influence of these factors on health outcomes. Further, gender disparities were evident, with females exhibiting higher stress levels and a greater prevalence of fibromyalgia. This pattern is consistent with existing literature, such as the work of Ruschak et al. (2023), which highlights that women are more susceptible to chronic pain conditions like fibromyalgia due to various factors.^26^ Additionally, Lakshmi and Prasanth (2018) demonstrated that women experience significant difficulties in achieving work-life balance due to the dual burden of professional and domestic responsibilities, compounded by sector-specific challenges and societal expectations.^27^ These findings align with our study, where the mean self-reported stress score among females was 6.7, significantly higher than the 5.6 reported by males (p = 0.002), further emphasizing the gender disparity in stress levels. While comparing different healthcare roles, the study finds that physicians reported higher stress levels than nurses, which aligns with findings by Shanafelt et al. (2012), who reported that physicians often face higher stress due to their critical decision-making roles and patient care responsibilities.^28^ Wallace, Lemaire, and Ghali (2009) discussed the significant stressors faced by physicians, including heavy workloads, emotional demands, and cognitive challenges, which negatively impact their well-being and the quality of healthcare.^29^ Our data shows that physicians, particularly attending physicians, had a mean stress score of 7.5, significantly higher than the 5.7 reported by nurses, demonstrating the increased stress burden in high-stakes specialties. These factors are likely influenced by the specific healthcare context in Abu Dhabi, where high patient loads and cultural expectations can exacerbate stress levels. In contrast to the findings of our study regarding workplace stressors like lack of control and recognition, Lemaire and Wallace (2017) suggested that supportive work environments and recognition can significantly reduce stress among healthcare professionals.^30^ This discrepancy could be due to differences in the study populations or the organisational cultures within different healthcare settings. The findings suggest that improving these factors could be key to mitigating occupational stress and its related health outcomes. Moreover, the influence of workplace factors can vary significantly across different clinical departments. For instance, our study revealed that healthcare professionals in high-pressure specialties such as Cardiology and Anaesthesia reported the highest stress scores, with means of 10 and 9, respectively, compared to lower levels observed in departments like Psychiatry and Paediatrics.

Several other health outcomes were found to be significantly correlated with stress in our study, compared to other healthcare professionals, nurses were most likely to report experiencing various of stress-related symptoms, including headache, waking up tired, fatigue, abdominal pain, trouble thinking, and depression (**Figure 3**). These associations are consistent with the existing literature, which links high levels of occupational stress to a range of physical and mental health conditions.^2,4^ The diverse range of health outcomes linked to stress necessitates the need for a comprehensive approach to managing occupational stress in healthcare settings.

Nonetheless, the cross-sectional nature of this study limits the ability to establish causality between occupational stress and fibromyalgia. Therefore, longitudinal studies are needed to explore this relationship further and to identify potential early intervention strategies. Additionally, more in-depth studies should examine the unique stressors faced by different healthcare roles and how demographic factors such as gender and age interact with these stressors. These studies should also consider variations in study design and measurement methods to better understand discrepancies between findings. Furthermore, several comorbidities such as anxiety, depression, diabetes, hypothyroidism, obesity, irritable bowel syndrome, migraine, vitamin D deficiency, and autoimmune disease, were identified among the study participants. These observations provide important insight into the broader health impact of occupational stress and highlight the need for comprehensive intervention addressing these overlapping health concerns. Another limitation of the study is the self-developed questionnaire, which was not tested formally for reliability and consistency. However, the pilot study testing was included, and the experts were reviewed to mitigate the limitation by ensuring the clarity and relevance of those questionnaires.

Moreover, the findings highlight the need for targeted interventions and policy changes in healthcare settings to address occupational stress and its impact on health outcomes. Implementing comprehensive stress management programs, improving workplace conditions, and providing tailored support for female healthcare professionals is crucial for reducing the high levels of stress and fibromyalgia observed. In line with these recommendations, participants in this study suggested specific strategies such as stress management training, improved communication, and more flexible work hours as effective measures to mitigate stress. Thus, healthcare institutions can significantly improve the well-being of their staff and enhance the quality of care provided to patients by addressing these factors. Future research should continue to explore the complex interplay between occupational stress and chronic health conditions to develop evidence-based strategies for improving the well-being of healthcare workers.

## CONCLUSIONS

Our study revealed a high prevalence of occupational stress and fibromyalgia among healthcare professionals in Abu Dhabi, with stress levels considerably higher among females, shift workers, and those in high intensity specialties such as Cardiology and Anaesthesia. The prevalence of fibromyalgia (28.3%) among this cohort is markedly higher than that observed in the general population, suggesting an association between occupational stress and fibromyalgia in healthcare settings. Gender disparities were also evident, with female healthcare professionals reporting higher stress levels and a greater prevalence of fibromyalgia. These results reveal the role that certain factors, such as gender, play in shaping occupational health outcomes. There is an urgent need for healthcare institutions in Abu Dhabi to implement comprehensive stress management programs, such as flexible scheduling, mental health support services, and organisational changes to alleviate these stressors.

## CONFLICT OF INTEREST

The authors declare no conflict of interest.

## AUTHOR CONTRIBUTIONS

All authors contributed to the final manuscript, and they meet all the four criteria of the International Committee of Medical Journal Editors. AA, NYA, HMA, and SHA: Literature screening and manuscript preparation. SA, MJH and SAA: review, analysis, writing and editing the final manuscript. All authors take full responsibility for the integrity and accuracy of all aspects of the work.

## STATEMENT OF ETHICS

This study was conducted in compliance with the 1964 Helsinki Declaration. The study was approved by SEHA Human Research Ethics Committee, on 23/10/2023 (Project number 295); informed consent was obtained from all the study participants.

## Figures and Tables

**Supplementary Table 1.** Perception of healthcare professionals regarding WSS.

**Conditions at work are unpleasant or sometimes even unsafe**	**Counts**	**% of Total**
**Never**	29	11.4
**Often**	34	13.4
**Rarely**	76	29.9
**Sometimes**	104	40.9
**Very often**	11	4.3
**I feel that my job is negatively affecting my physical or emotional well-being.**		
**Never**	29	11.4 %
**Often**	58	22.8 %
**Rarely**	47	18.5 %
**Sometimes**	95	37.4 %
**Very often**	25	9.8 %
**I have too much work to do an/or too many unreasonable deadlines.**		
**Never**	27	10.6 %
**Often**	49	19.3 %
**Rarely**	61	24.0 %
**Sometimes**	94	37.0 %
**Very often**	23	9.1 %
**I find it difficult to express my opinions or feelings about my job conditions to my superiors.**		
**Never**	35	13.8 %
**Often**	45	17.7 %
**Rarely**	68	26.8 %
**Sometimes**	76	29.9 %
**Very often**	30	11.8 %
**I feel that job pressures interfere with my family or personal life.**		
**Never**	32	12.6 %
**Often**	48	18.9 %
**Rarely**	40	15.7 %
**Sometimes**	87	34.3 %
**Very often**	47	18.5 %
**I have adequate control or input over my work duties.**		
**Never**	9	3.5 %
**Often**	91	35.8 %
**Rarely**	35	13.8 %
**Sometimes**	91	35.8 %
**Very often**	28	11.0 %
**I receive appropriate recognition or rewards for good performance.**		
**Never**	26	10.2 %
**Often**	53	20.9 %
**Rarely**	79	31.1 %
**Sometimes**	90	35.4 %
**Very often**	6	2.4 %
**I am able to utilitze my skills and talents to the fullest extent at work.**		
**Never**	4	1.6 %
**Often**	87	34.3 %
**Rarely**	40	15.7 %
**Sometimes**	84	33.1 %
**Very often**	39	15.4 %
**Negative impact and training participation**		
**Thought of quitting job**	137	54%
**Never attended stress training**	221	87%

**Supplementary Table 2.** Linear Regression analysis for significant factors for stress.

**Predictor**	**Estimate**	**SE**	**t**	**p**
Intercept	7.3066	0.825	8.8526	< .001
**Gender:**				
Female – Male	1.2492	0.329	3.7969	**< .001**
**Age:**				
30–39 – 20–29	−1.0077	0.552	−1.827	0.069
40–49 – 20–29	−0.0234	0.723	−0.0324	0.974
50–59 – 20–29	0.3293	0.831	0.3962	0.692
60+ – 20–29	−2.062	1.473	−1.4002	0.163
**Marital status:**				
Divorced – Single	−0.138	1.03	−0.1339	0.894
Married – Single	−0.3929	0.314	−1.2496	0.213
Widow/widower – Single	−2.7089	2.069	−1.3091	0.192
**Career Level/status:**				
GP (without formal residency training) – Consultant	1.6916	1.01	1.6746	0.095
Nurse – Consultant	1.9227	0.554	3.4735	**< .001**
Residents – Consultant	0.8346	0.663	1.2588	0.209
Specialist – Consultant	0.5793	0.59	0.9814	0.327
**Nationality:**				
Non Emirati – Emirati	−0.1099	0.415	−0.2648	0.791
**Ethnicity:**				
Asian – Arab	0.3611	0.425	0.8507	0.396
Black – Arab	0.7901	1.23	0.6424	0.521
Others – Arab	−1.0091	0.793	−1.2719	0.205
White – Arab	0.0945	0.977	0.0967	0.923
**Work turns:**				
Shifts/Rotatory – Fixed hours	0.7231	0.279	2.5902	**0.01**
**Smoking status:**				
Current smoker – Non-smoker	1.0473	0.74	1.4155	0.158
Ex-smoker – Non-smoker	1.8015	0.713	2.527	**0.012**
**Duration of working**				
10–15 years – 5–10 years	−0.8627	0.46	−1.8772	0.062
Less than 5 years – 5–10 years	−0.5991	0.411	−1.4562	0.147
More than 15 years – 5–10 years	−0.5047	0.571	−0.8843	0.377

**Supplementary Table 3.** Logistic regression analysis factors affecting Fibromyalgia.

**Logistic regression analysis**					**95% Confidence Interval**	
**Predictor**	**Estimate**	**Z**	**p**	**Odds ratio**	**Lower**	**Upper**
Intercept	−3.2842	−2.03171	0.042	0.0375	0.00158	0.891
**Gender:**						
Female – Male	1.6359	3.02121	**0.003**	**5.1341**	**1.77648**	**14.838**
**Age:**						
30–39 – 20–29	−0.0769	−0.12849	0.898	0.926	0.28661	2.992
40–49 – 20–29	0.1909	0.28069	0.779	1.2103	0.31925	4.588
50–59 – 20–29	−0.5963	−0.68115	0.496	0.5508	0.09903	3.064
60+ – 20–29	−14.8276	−0.00664	0.995	3.63E-07	0	Inf
**Marital status:**						
Married – Divorced	0.8297	0.62037	0.535	2.2926	0.1667	31.529
Single – Divorced	1.2816	0.96147	0.336	3.6025	0.26422	49.119
Widow/widower – Divorced	−14.3681	−0.00363	0.997	5.75E-07	0	Inf
**Career Level/status**						
GP (without formal residency training) – Consultant	−0.0367	−0.02717	0.978	0.964	0.06824	13.617
Nurse – Consultant	0.3704	0.53892	0.59	1.4483	0.37659	5.57
Residents – Consultant	−0.2299	−0.28171	0.778	0.7946	0.16051	3.934
Specialist – Consultant	0.417	0.57109	0.568	1.5173	0.36275	6.347
**Nationality:**						
Non Emirati – Emirati	−0.4506	−0.90749	0.364	0.6373	0.24082	1.686
**Ethnicity:**						
Asian – Arab	−0.8731	−1.67678	0.094	0.4177	0.15052	1.159
Black – Arab	0.4913	0.34557	0.73	1.6344	0.10075	26.515
Others – Arab	−17.3702	−0.01379	0.989	2.86E-08	0	Inf
White – Arab	1.1938	1.0044	0.315	3.2996	0.32118	33.898
**Work turn:**						
Shifts/Rotatory – Fixed hours	0.76	2.18215	**0.029**	**2.1383**	**1.08046**	**4.232**
**Smoking status:**						
Current smoker – Non-smoker	1.7242	1.9844	**0.047**	**5.6079**	**1.02146**	**30.788**
Ex-smoker – Non-smoker	0.6675	0.72127	0.471	1.9493	0.31781	11.956

**Table:** 

**I. Indicate the level of severity over the past week by checking the appropriate box**						
			0	1	2	3
			No problem	Mild	Moderate	Severe
Fatigue						
Trouble thinking or remembering						
Waking up tiered (unrefreshed)						
**II. During the past 6 months have you had any of the following symptoms?**						
			Yes (1)		No (0)	
Pain or cramps in the lower abdomen						
Depression						
Headache						
III. **Joint/body pain: please indicate below if you have had pain or tenderness over the past 7 days in each of the areas listed below. (image is provided below to clarify the site)**						
	Yes	No				
Shoulder, left						
Shoulder, right						
Hip, left						
Hip, right Upper						
Αrm, left						
Upper arm, right						
Lower arm, left						
Lower arm, right						
Upper leg, left						
Upper leg, right						
Lower leg, left						
Lower leg, right						
Jaw, left						
Jaw, right						
Chest						
Abdomen						
Lower back						
Upper back						
Neck						
OTHER SYMPTOMS that you have experienced over the past week?						
Overall, were the symptoms (pain/tendor points) listed above generally present for at least 3 months?*						
Yes						
No						

## Occupational Stress among Healthcare Workers - Survey

Dear healthcare workers,

You are invited to participate in our research study titled “Occupational Stress among Healthcare Workers (Physicians/Nurses) in Abu Dhabi.” This survey aims to gather insights to enhance the healthiness of the work environment. Rest assured, the information you provide will solely be used for research purposes and will not be utilized for any other intent. Your participation in this survey is entirely voluntary, and responding to all questions should take approximately 5 minutes. Your responses are confidential. Before proceeding, please indicate your choice below. By selecting “Agree,” you confirm that you have read the above information and willingly agree to participate in the survey. *

Agree Disagree

### Personal information

Gender *

Female
Male

Age *

20–29
30–39
40–49
50–59
60+

Nationality *

Emarati
Non Emarati

Ethnicity *

Arab
Asian
White
Black
Others

Marital status *

Single
Married
Divorced
Widow/widower

Career Level/status? *

GP (without formal residency training)
Residents
Specialist
Consultant
Nurse

How many people are depending on you financially? *

Hospital *

Govermental
Private

Clinical department *

Anesthesia
Cardiology
Dermatology
ENT
ER/Emergency
Family medicine
Gastroenterology
General Internal Medicine
General surgery
Geriatrics
Hematology/Oncology
ICU
Infectious disease
Neurology
Nephrology
Neurosurgery
ObGYN
Opthalmology
Orthopedics
Pediatrics
Psychiatry
Plastic surgery
Pathology
Physiotherapy and rehabilitation
Rheumatology
Respiratory
Radiology
Respiratory Therapist
Thoracic surgery
Urology
Medical nurse
Surgical nurse
Emergency-nurse
Nurse-Midwives
Others

Work turn *

Fixed hours
Shifts/Rotatory

Smoking status *

Current smoker
Non-smoker
Ex-smoker

How long have you been working in the healthcare system in Abu Dhabi Emirate? *

Less than 5 years
5–10 years
10–15 years
More than 15 years

Please mention if you have any of below medical conditions. Yes No

Anxiety
Cancer
Cardiovascular disease
Chronic lung disease
Chronic renal disease
Depression
Diabetes
Dyslipidemia
Hypertension
Hypothyroidism
Obesity
Irritable bowel syndrome
Migraine
Vitamin D deficiency
Autoimmune related conditions (rheumatoid arthritis, psoriatic arthritis, ankylosing spondylitis, lupus, etc)
Occupational stress

Thinking about your current job, how often does each of the following statements describe how you feel? (Q1–Q8)

1. Conditions at work are unpleasant or sometimes even unsafe *
  Never
  Rarely
  Sometimes
  Often
  Very often
2. I feel that my job is negatively affecting my physical or emotional well-being. *
  Never
  Rarely
  Sometimes
  Often
  Very often
3. I have too much work to do an/or too many unreasonable deadlines. *
  Never
  Rarely
  Sometimes
  Often
  Very often
4. I find it difficult to express my opinions or feelings about my job conditions to my superiors. *
  Never
  Rarely
  Sometimes
  Often
  Very often
5. I feel that job pressures interfere with my family or personal life. *
  Never
  Rarely
  Sometimes
  Often
  Very often
6. I have adequate control or input over my work duties.*
  Never
  Rarely
  Sometimes
  Often
  Very often
7. I receive appropriate recognition or rewards for good performance. *
  Never
  Rarely
  Sometimes
  Often
  Very often
8. I am able to utilitze my skills and talents to the fullest extent at work. *
  Never
  Rarely
  Sometimes
  Often
  Very often

Quantify the level of stress before joining the institution out of 10 (Excluding Internship) *

Quantify the level of stress after joining the institution (excluding the COVID 19 pandemic era) *

What is the main source of your stress? *

Work
Home
Both
Other

Have you ever thought of quitting your job because of work related-stress? *

Yes
No

Do you think occupational stress can be minimized? *

Yes
No

How can occupational stress be minimized? Tick the top five most important from your point of view *

Better Team Communication
Collaborative Workspaces
Employee Recognition Programs
Flexible Work Arrangements
Health and Wellness Programs
Incorporating psychological assistance within the occupation
Increase annual leave
Increase salary
Improving working hours
Leadership Development Programs
Managment Development
More CME hours
Proper communication between workers
Regular meeting with Admin team
Regular departmental meeting
Stress managment Training
Yoga/Stretch Breaks

Have you attended the occupational stress workshop/programs (e.g Relaxation Clinic and OR hotline service) provided by the hospital? *

Yes
No

If yes, how effective was it in helping you manage stress? *

Very effective
Somewhat
A little
Not at all

If you did not attend, why?

Timing was not suitable
I was not informed about it
Not interested
Not available at my hospital

Are there any strategies which could be adopted to prevent or reduce stress among staff?

## Fibromyalgia Survey Questionnaire (FSQ)

Assess the key symptoms of fibromyalgia
